# Distribution of absorbed photons in the tunneling ionization process

**DOI:** 10.1038/s41598-021-83453-0

**Published:** 2021-02-17

**Authors:** I. A. Ivanov, Kyung Taec Kim

**Affiliations:** 1 Center for Relativistic Laser Science, Institute for Basic Science, Gwangju, 61005 Korea; 2 Department of Physics and Photon Science, GIST, Gwangju, 61005 Korea

**Keywords:** Atomic and molecular interactions with photons, Quantum mechanics

## Abstract

We describe a procedure that allows us to solve the three-dimensional time-dependent Schrödinger equation for an atom interacting with a quantized one-mode electromagnetic field. Atom-field interaction is treated in an ab initio way prescribed by quantum electrodynamics. We use the procedure to calculate probability distributions of absorbed photons in the regime of tunneling ionization. We analyze evolution of the reduced photon density matrix describing the state of the field. We show that non-diagonal density matrix elements decay quickly, as a result of the decoherence process. A stochastic model, viewing ionization as a Markovian birth-death process, reproduces the main features of the calculated photon distributions.

## Introduction

Tunneling ionization is a process which can be pictured as a non-resonant absorption of a large number of photons from a driving electromagnetic field. The strong field approximation (SFA) theory, developed by Keldysh^1^, defines the tunneling regime of ionization as the regime characterized by the values of the Keldysh parameter $\gamma =\omega \sqrt{2|\varepsilon _0|}/E \lesssim 1$ (here $\omega$, *E* and $|\varepsilon _0|$ are the frequency, field strength and ionization potential of the target system). This theory and its subsequent developments^2–8^ provide the basis for understanding various phenomena accompanying the process of the tunneling ionization, such as above-threshold ionization or high harmonic generation.

The approaches we mentioned above consider electromagnetic field and the field-atom interaction in an entirely classical way. A quantum description of light and light-matter interaction is provided by quantum electrodynamics (QED)^9^. In the present manuscript, we describe an approach allowing us to solve the time-dependent Schroödinger equation (TDSE) numerically for an atom interacting with an electromagnetic field, with field-atom interaction described in the framework of QED. The necessity and usefulness of such a description is obvious for very high field strengths which can already be obtained in laboratories. Development of laser techniques^10–14^ has allowed us to reach intensities of laser radiation of the order of $10^{23}$ W/cm$^2$, and the attainment of yet higher intensities of the order of $10^{26}$ W/cm$^2$^15^ can be expected in the near future. At these intensities, a plethora of new phenomena, such as various effects due to the electron spin^16^, quantum radiation reaction effects^17^, and many others^15,18^ opens up for experimental study.

It is, in part, the possibility of studying these phenomena which prompted our interest in developing the present procedure, based on the QED description of the photon field. What will interest us below, however, are not the effects appearing at very high field strengths. We will consider weaker fields with intensities of the order of $10^{14}-10^{15}$ W/cm$^2$. Even for such field intensities the description of the atomic or molecular photo-ionization based on the non-relativistic TDSE may have its limitations. This fact has been realized since the pioneering work by Reiss^19^, where it was shown that in the tunneling regime of ionization relativistic effects may prove quite significant. In particular, relativistic non-dipole effects are visible in the experimentally observed photo-electron spectra^20^ for the infrared (IR) laser fields of the intensity of the order of $10^{14}$ W/cm$^2$. These non-dipole effects in the tunneling regime of ionization have been studied experimentally^20,21^, and theoretically^16,22–28^. Effects related to the electron spin, which cannot be described by the non-relativistic TDSE, have also been observed. A strong spin asymmetry is present in the spectra of the above threshold ionization process (ATI)^16,29^. The free-electron lasers (FEL)^12–14^ offer a possibility of the experimental study of the relativistic effects in the domain of high photon energies, where the non-dipole effects due to finite photon momentum can be expected to be particularly important^30,31^.

The relativistic and spin effects can be studied using the relativistic generalizations of the SFA theory^7,16,19,22,25,28^. Alternatively, for not very high field strengths, one can use perturbative approach, by adding the terms describing relativistic interactions to the non-relativistic Hamiltonian^23,24^ and solving the resulting TDSE numerically. Treatment of higher field strengths, where electron acquires relativistic velocities, but electromagnetic fields can can still be considered classically, is possible with the use of the approaches based on the numerical solution of the time-dependent Dirac equation (TDDE)^31–34^.

QED offers a natural framework for taking into account relativistic and electron spin effects. There is, however, another, equally important aspect of the QED which is related to the fact that in this theory electrons and photons are considered as quantum fields. In the present work we will exploit a possibility which the QED approach to the description of the photon fields offers: the possibility to “count” the photons, i.e., the possibility to study statistics and distributions of photons, tracking evolution of the state of the field during the process of atom-field interaction. More specifically, we will consider the process of tunneling ionization. As we will see, analyzing the photon distributions may reveal some interesting features of the process.

Atomic units (a.u.) with $m=e=\hbar =1$ (here *e* and *m* are electron charge and mass) are used throughout the paper. The speed of light in these system of units is $c\approx 137.036$ a.u.

## Results

### Theoretical model

We will first outline briefly the procedure we employ to describe an atom interacting with a quantized electromagnetic field. The quantized vector potential can be written as^9,35^:

1 $$\begin{aligned} \hat{{{\varvec{A}}}}({{\varvec{r}}},t)=\sum \limits _{{{\varvec{k}}},\lambda } \sqrt{2\pi c^2\over \omega V} \left( {{\varvec{e}}}_{{{\varvec{k}}},\lambda } \hat{a}_{{{\varvec{k}}},\lambda } e^{-iwt+i{{\varvec{k}}}\cdot {{\varvec{r}}}} + h.c \right) \ , \end{aligned}$$

where it is assumed that the electromagnetic field is quantized in a finite volume *V*, and $a_{{{\varvec{k}}},\lambda }$ are the photon annihilation operators. The combined Hilbert space of the system atom+ field is the tensor product ${\mathcal{H}}_\text{atom}\otimes {\mathcal{H}}_\text{field}$. Here ${\mathcal{H}}_\text{atom}$ and ${\mathcal{H}}_\text{field}$ are electron and photon sectors of the Hilbert space, respectively. We will employ the well-known fact that the photon Hilbert space is spanned by the Fock states $|N\rangle$- the eigenstates of the operator $\hat{N}_{{{\varvec{k}}},\lambda }=\hat{a}^{\dag }_{{{\varvec{k}}},\lambda }\hat{a}_{{{\varvec{k}}},\lambda }$ of the number of photons in the mode ${{\varvec{k}}},\lambda$. We will consider below only one mode of the field, corresponding to linear polarization in the *z*-direction and a particular photon frequency $\omega =0.057$ a.u. (wavelength of 800 nm). We will omit, therefore, subscripts ${{\varvec{k}}},\lambda$ in all the formulae below. Using the basis of the Fock states, the matrix elements of the photon operators in Eq. (1) assume the well-known form^36^:

2 $$\begin{aligned} \langle N-1|a|N\rangle= & {} \sqrt{N} \nonumber \\ \langle N+1| a^{\dag }|N\rangle= & {} \sqrt{N+1} \ , \end{aligned}$$

while all other matrix elements have zero values. The state of the combined system atom+field at the initial moment of time, $t_0=0$ a.u., is assumed to be a tensor product $\phi _0 \otimes |N_0\rangle$ of the ground atomic state $\phi _0$ and the Fock state $|N_0\rangle$. Subsequent evolution of the system is governed by the time-dependent Schrödinger equation (TDSE)^9^ (we use velocity gauge to describe atom-field interaction):

3 $$\begin{aligned} i{\partial |\Phi (t)\rangle \over \partial t}= \left( \hat{H}_\text{atom} + {1\over c}{\hat{{{\varvec{A}}}}}\hat{{{\varvec{p}}}} + {1\over 2c^2}{\hat{{{\varvec{A}}}}}^2 \right) |\Phi (t)\rangle \ , \end{aligned}$$

where $\displaystyle \hat{H}_\text{atom}$ is atomic Hamiltonian. We do not have to include the field Hamiltonian in Eq. (3) because the vector potential in Eq. (1) is time-dependent, i.e., the representation we use in Eq. (3) is the Schrödinger representation for the atomic operators and interaction representation for the field operators. This representation can be obtained from the Schrödinger picture, in which neither atomic nor field operators depend on time, by means of the unitary transformation $\exp {\left\{ -i\hat{H}_\text{field}t\right\} }$ generated by the field Hamiltonian $\hat{H}_\text{field}$.

We use a non-relativistic form, $\displaystyle \hat{H}_\text{atom}= {\hat{{{\varvec{p}}}}^2\over 2}+V(r)$, of the atomic Hamiltonian, with the short-range atomic potential $V(r)=-1.903 e^{-r}/r$. This potential supports only one bound state of $s-$ symmetry with ionization potential $|\varepsilon _0|=0.5$ a.u. Though our computational procedure can be applied equally well for the case of the Coulomb potential, we choose a short-range atomic potential with only one bound state to concentrate fully on ionization by excluding all effects due to excitation processes. Another assumption we make is the dipole approximation, which consists in neglecting the spatial dependence of the vector potential in Eq. (1). Both these assumptions are easily justified for the moderate field intensities (of the order of several units of $10^{14}$ W/cm$^2$) that we consider below. A short explanation of what we mean by the light intensity may be appropriate here. As is well-known, for the Fock state of the field, the expectation values of the field operators (e.g., the vector potential (1)) are zero. To relate the photon number *N* to the observable effects, we can use instead the cycle-averaged expectation value of the Poynting operator, which in the Fock state $|N\rangle$ is^36^ $\displaystyle \omega c N/V$. The cycle-average for the Poynting vector, computed for the classical monochromatic linearly polarized wave wave ${{\varvec{E}}}_0 \cos {\omega t}$, is, on the other hand: $\displaystyle cE_0^2/(8\pi )$. From the point of view of the time-averaged flux of energy, the Fock state $|N_0\rangle$ is, therefore, equivalent to a monochromatic wave with $E_0=\sqrt{8\pi \omega N_0/V}$. We will call $E_0$ defined in this way the ’equivalent field strength’.

To solve evolution equation (3) numerically, we use the completeness of the Fock states in ${\mathcal{H}}_\text{field}$, and expand the time dependent wave-function of the atom+field system as:

4 $$\begin{aligned} |\Phi (t)\rangle = \sum \limits _{N=N_0-n1}^{N_0+n2} |f_N(t)\rangle \otimes |N\rangle \ , \end{aligned}$$

where $|f_N(t)\rangle$ are vectors from the atomic Hilbert space ${\mathcal{H}}_\text{atom}$, and the parameters $n_1$, $n_2$ define the range of the Fock states we need to keep to ensure convergence of the expansion (4). Details of the procedure we use to solve the TDSE (3) using the expansion (4) are given in the Section “Methods” below. We solve the TDSE for the interval of time (0, *MT*), where $T=2\pi /\omega$ is an optical cycle corresponding to the driving frequency $\omega =0.057$ a.u. For the majority of the calculations we report below, we used $M=12$. That this choice of *M* is adequate for the purposes of the present work is shown in the section “Methods” below.

We are primarily interested in the evolution of the state of the electromagnetic field on the interval of pulse duration. The state of the field can be described by the reduced density matrix $\rho _F(t)$^36^, which can be computed from the density matrix $\hat{\rho }(t)=|\Phi (t)\rangle \langle \Phi (t)|$ of the complete atom+field system obtained from the solution $|\Phi (t)\rangle$ of the TDSE, by taking a partial trace with respect to atomic variables. For the $|\Phi (t)\rangle$ represented as an expansion (4), the partial trace can be easily computed, giving the following expression for the reduced density matrix describing the state of the field:

5 $$\begin{aligned} \hat{\rho }_F(t)= \mathrm{Tr}_\text{atom} |\Phi (t)\rangle \langle \Phi (t)|= \sum _{N_1,N_2} \langle f_{N2}(t)|f_{N1}(t)\rangle |N_1\rangle \langle N_2| \ , \end{aligned}$$

where $\langle f_{N2}(t)|f_{N1}(t)\rangle$ is a scalar product of the vectors $f_N(t)$ from the atomic Hilbert space ${\mathcal{H}}_\text{atom}$ occurring in the expansion (4). Matrix elements of $\hat{\rho }_F(t)$ in the basis of the Fock states can, therefore, be easily computed once the TDSE equation (3) is solved. The diagonal matrix elements $\hat{\rho }_F^{NN}(t)$ then give us the probabilities $P_N(t)$ of observing the field in a state with *N* photons.

We will be interested below in the process of absorption of photons. For the field initially in the Fock state $|N_0\rangle$, the number of photons is fixed and the projection of the wave-function of the system at time *t* on the Fock state $|N_1\rangle$ directly gives us the probability of absorption or emission of $N_1-N_0$ photons. In the case of the initial Fock state of the field that we consider in the present work, the matrix element $\hat{\rho }_F^{{N_0-n},{N_0-n}}$ (where *n* is a positive integer) give us, therefore, the probability $P_n$ of absorption of *n* photons. From the point of view of experimental studies of photon absorption dynamics, a more natural choice would be a coherent state of the field. This choice entails a difficulty, however. For the coherent state the number of photons is not fixed and we can specify only the expectation value $N_0$ of the number of photons. The dispersion of the number of photons is proportional to $N_0^{1\over 2}$^36^ and can be much larger than the number of photons absorbed in the process of ionization. This makes the definition of the number of absorbed photons less straightforward for a coherent state. It is the possibility of the direct interpretation of the $\hat{\rho }_F^{{N_0-n},{N_0-n}}$ as the absorption probabilities which motivated our choice of the Fock state of the field as the initial state. We can expect, however, that the general features of the ionization dynamics which we describe below, will be valid in the case of ionization driven by the field in a coherent state. The phase of the field in a Fock state is completely undefined. It is known^37^ that the effect of the undefined field phase on the density matrices can be described as a suitable average of density matrices obtained for coherent states with different phases of the field. Therefore, we can expect this effect to vanish for long enough pulses. That this is indeed the case was shown, e.g., in^38^ by comparing electron spectra obtained for the case of ionization driven by the field in a coherent state and in a Fock state of the same effective field strength.

**Figure 1:**
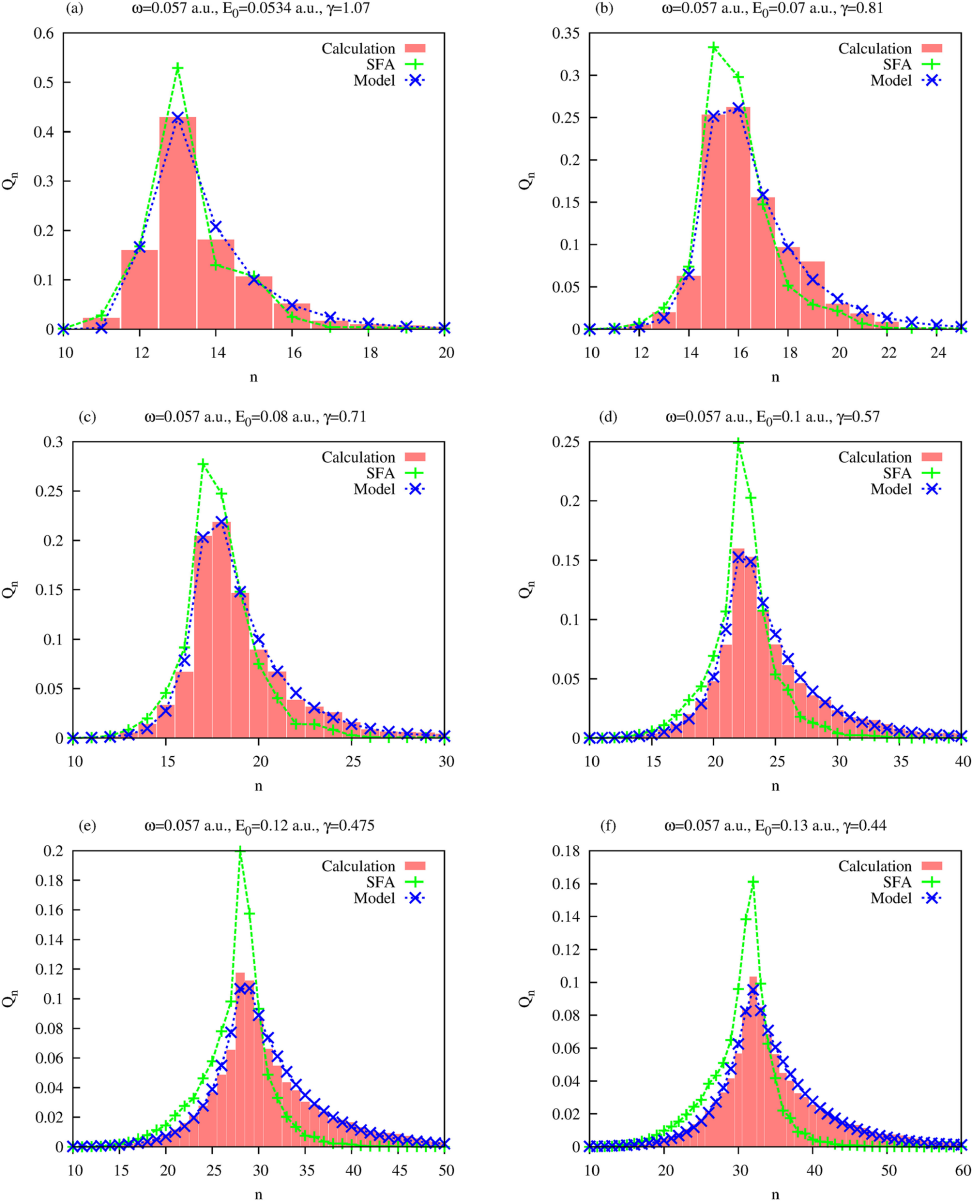
Distributions of absorbed photons, $Q_n$. Also shown are SFA distributions obtained using Eq. (17), and distributions obtained using the stochastic model based on Eqs. (8) and (9).

More convenient for the study of the absorption process is the normalized probability distribution $Q_n$. This distribution is a conditional probability of absorbing *n* photons from the field provided at least one photon has been absorbed, and it coincides with $P_n$ for $n\ge 1$ up to a normalization factor which ensures that $\sum \limits _{n=1}^{n=+\infty } Q_n=1$. The results for the probability distribution of absorbed photons, $Q_n$, that we obtain by solving the TDSE (3) and using Eq. (5) are shown in Fig. 1. For comparison, we also show in the Figure the results we obtain for $Q_n$ using the SFA (the details of this calculation are given in the Section “Methods”), and the results we obtain using a stochastic model of the ionization process we present below.

### Stochastic model of strong field ionization

To understand the main features of the distributions, $Q_n$, in Fig. 1, and get a better insight into the photon absorption for the strong field ionization process, we report below a study of the dynamics of the process. This study is based on an analysis of the reduced density matrix $\hat{\rho }_F(t)$ as a function of time on the interval of the pulse duration. The exact equation governing evolution of the reduced density matrix can be obtained by taking a partial trace with respect to the atomic variables of the von-Neumann equation describing quantum evolution of the atom+field system:

6 $$\begin{aligned} i{\partial \over \partial t}\hat{\rho }_F(t)= {\mathrm{Tr}}_\text{atom} \left[ {\hat{H}(t)},{\hat{\rho }(t)}\right], \end{aligned}$$

where $\hat{H}(t)$ and $\hat{\rho }(t)$ are the Hamiltonian and the density matrix of the system atom+field, respectively. Eq. (6) and the initial condition $\hat{\rho }(0)=|N_0\rangle \langle N_0|\otimes |\phi _0\rangle \langle \phi _0|$ (here $|N_0\rangle$ and $|\phi _0\rangle$ are initial states of field and atom) determine, in principle, subsequent evolution of the reduced photon density matrix. This equation is exact but can hardly be solved in practice for the system we are presently studying. Various simplifications of the general equation for the reduced density matrix, the so-called ’master equations’, describing evolution of a subsystem interacting with the environment (often called the ’reservoir’ in the literature) are known^39^. These approximations are usually based on the assumption that the evolution of the reduced density matrix is Markovian^39^, that is the process has no memory and its evolution for $t>t_0$ is defined by the state at $t_0$. In this case the general form of the master equation in the so-called Lindblad form^40^ can be derived.

#### Atom-field interaction as an example of decoherence phenomenon

**Figure 2:**
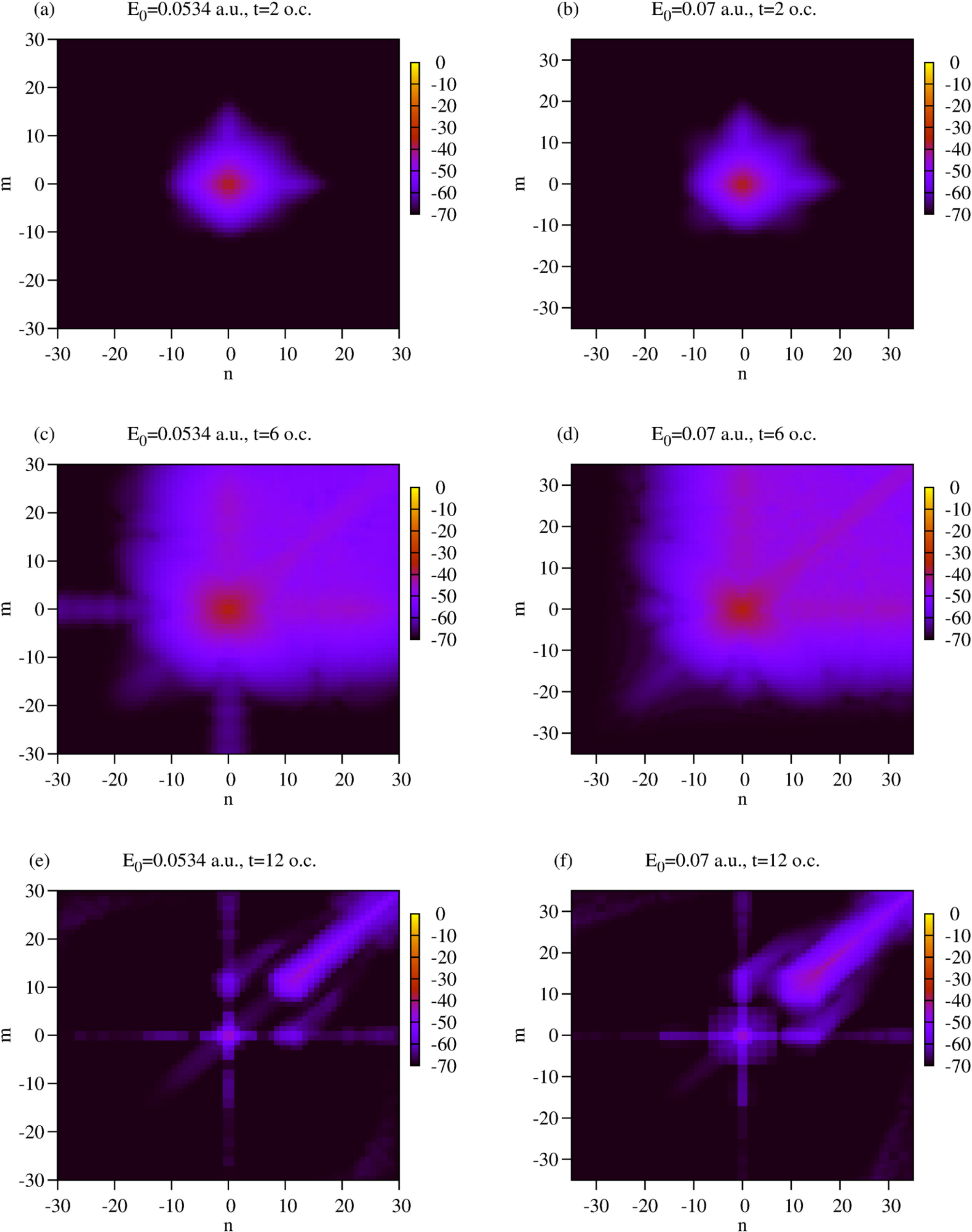
Absolute values of the reduced density matrix elements $\hat{\rho }_F^{{N_0-n},{N_0-n}}$. Logarithmic scale is used.

In the problem we are presently considering, the subsystem we are interested in is the electromagnetic field. Atom plays the role of the environment. That this separation may be meaningful and useful can be seen from Fig. 2, which shows absolute values of the reduced density matrix elements $\hat{\rho }_F^{{N_0-n},{N_0-n}}$ as functions of time (measured in the units of the optical cycles).

One observes from the Fig. 2 that the density matrix elements $\hat{\rho }_F^{{N_0-n},{N_0-n}}$ with negative $n_1$, $n_2$ (corresponding to the emission of photons) have negligible values. This is, of course, expected for the long pulses we consider presently. More interestingly, we observe the progressive decay of the non-diagonal elements of the density matrix for positive $n_1$, $n_2$. For the time $t=12$ o.c, for both field strength values shown in Fig. 2, matrix elements are predominantly concentrated along the main diagonal with positive $n_1$, $n_2$. Such behavior is, in fact, typical for the density matrix of a subsystem interacting with the environment, and is a manifestation of the decoherence process^41^. The decoherence process has been invoked^41–44^ to clarify the measurement problem and to understand the transition from the quantum to classical description in quantum mechanics.

Decay of the non-diagonal elements of the reduced density matrix can be understood as follows. According to Eq. (5), matrix elements of the reduced density matrix are the overlaps $\langle f_{N2}(t)|f_{N1}(t)\rangle$ of the corresponding states of the environment (states of the atom in our case). With increasing time, the environment states $|f_{N}(t)\rangle$ with different *N* become, progressively, more and more separated in energy, and thus, their overlaps tend to zero. The state (4) of the atom+ field system evolving from the initial product state $|\phi _0\rangle \otimes |N_0\rangle$ is an entangled state. With the atomic vectors $|f_{N}(t)\rangle$ becoming approximately orthogonal with time, a measurement performed on an atom which finds the atom in the state $|f_{N}(t)\rangle$, allows us, therefore, to state with certainty that the field is in the corresponding state $|N\rangle$. For large enough times, therefore, the environment (i.e. the atom) carries complete information about the system (field). This is, indeed, the decoherence process at work, as described, e.g., in^43^. Thus, we see that by picturing the field as a system, and the atom as the environment, we encounter an interesting example of the decoherence process which manifests itself through the decay of the non-diagonal matrix elements, computed in the basis of Fock states, of the reduced photon density matrix.

#### Evolution of diagonal elements of the reduced density matrix

In the previous section, we saw that, due to the decoherence process, the non-diagonal matrix elements of the reduced photon density matrix become small for large enough time. We now turn our attention to the diagonal elements of the reduced density matrix. As we mentioned above, under the assumption of the Markovian character of the process, the master equation describing evolution of the reduced density matrix can be written using the Lindblad operators, which in our case can be represented as matrices with dimension equal to the number of photon states we consider. For the problem of a one-mode electromagnetic field in a cavity interacting with a bath (which may be, e.g., the phonons in the material of the walls of the cavity), the master equation can be further simplified to give a relation of the form^39^:

7 $$\begin{aligned} {dP_n(t)\over dt}= \lambda _{n-1}P_{n-1}(t)+ \mu _{n+1}P_{n+1}- (\lambda _{n}+ \mu _{n})P_n(t) \ , \end{aligned}$$

where, using the notation we employed above, $P_n(t)= \hat{\rho }_F^{{N_0-n},{N_0-n}}$ are diagonal elements of the reduced photon density matrix. For the problem considered in^39^ the explicit expressions for the coefficients $\lambda _n$ ans $\mu _n$ in this equation can be given. We cannot use those particular expressions, however, since our problem differs somewhat from the one considered in^39^, where the environment was considered to remain in thermal equilibrium during the process. This would certainly not be the case in our problem, where the environment (the atom) is not in an equilibrium state for the whole interval of the pulse duration. However, we can preserve the general structure of Eq. (7) as the master equation describing the evolution of the density matrix in our problem. Indeed, Eq. (7) is a Kolmogorov equation^36,45^ describing the so-called ‘birth-death processes’^45–47^, and is, therefore, sufficiently general for our purposes. More detailed justification of the possibility to use Eq. (7) for the description of the evolution of the diagonal photon density matrix elements is given in the section “Methods” below.

The ’birth-death processes’ are continuous-time Markov chains^45^ in which only jumps to the neighboring states are allowed at small intervals of time. The meaning of the parameters $\lambda _{k}$ and $\mu _k$ in Eq. (7) in our case is the rate of absorption ($\lambda _{k}$) or emission of a photon ($\mu _k$) in a photon state, where *k* photons have already been absorbed. Using Eq. (7) as the master equation for our problem, we thus assume a Markovian character for the process. We can use the Kolmogorov equation (7), not only for the probability distribution $P_n$, but also for the normalized distribution $Q_n$ of the number of absorbed photons. One can see that if $P_n(t)$ obeys Eq. (7), evolution of $Q_n(t)$ for large enough times is also described by an equation of the type (7). Indeed, by definition for $n\ge 1$: $Q_n(t)=P_n(t)/C$, where *C* is the normalization factor equal to the total probability of absorption of at least one photon, which for long enough pulses should be equal to the total ionization probability. In the field regime we consider, the total ionization probability, in turn, is proportional to time. Therefore the constant $C\propto t$. We have, therefore, $Q_n(t)\propto P_n/t$. Substituting this expression into Eq. (7) and neglecting terms of the order of $1/t^2$, we obtain a Kolmogorov type equation (7) for $Q_n(t)$.

What will interest us now is the steady state solution of Eq. (7), i.e., the solution to which solutions $Q_n(t)$ of the Kolmogorov equation (7) tend in the limit $t\rightarrow \infty$. Such a solution represents the equilibrium state; in our case it is the equilibrium between the atom and the field reached at the end of the ionization process. A sufficient condition for the steady state distribution to exist is $|{\lambda _{k-1}/ \mu _{k}}|<1$ for all *k* greater than some $k_0$. The steady state solution, if it exists, can be obtained from Eq. (7) by equating time-derivatives on the left hand side to zero and solving the recurrence relation thus obtained. The results reads^48^:

8 $$\begin{aligned} Q_{n}= \mathrm{const} \times \prod \limits _{k=1}^{n} {\lambda _{k-1}\over \mu _{k}} \ , \end{aligned}$$

where the constant can be determined from the normalization condition $\displaystyle \sum \limits _{n=1}^{\infty } Q_n=1$.

**Figure 3:**
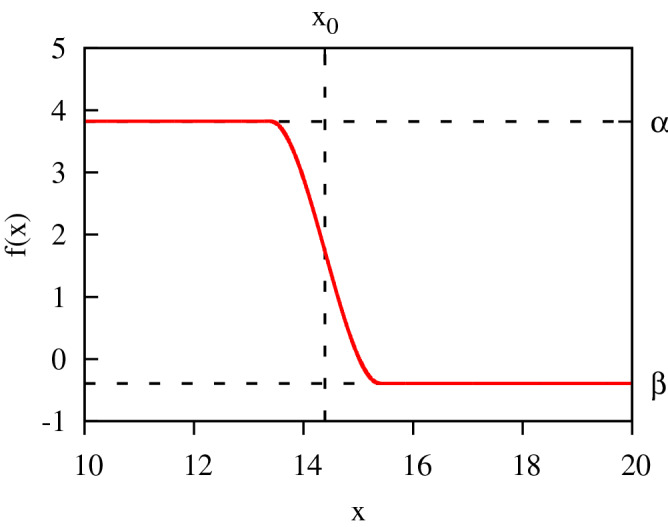
Typical behavior of the function *f*(*x*) in Eq. (9).

As for the rate coefficients $\lambda _n$ ans $\mu _n$ in Eq. (7), we will try to find a set of them having as simple a form, and as few free parameters as possible. As one can see from Eq. (8), it is the ratio ${\lambda _{k-1}/ \mu _{k}}$ which determines $Q_n$. We represent this ratio as ${\lambda _{k-1}/ \mu _{k}}= 1+ f(k)$, and we use the following trial form for the function *f*(*k*):

9 $$\begin{aligned} f(x)= \begin{Bmatrix} \alpha ,&\quad x < x_0-1 \\ q(x),&\quad x \in (x_0-1,x_0+1) \\ \beta ,&\quad x > x_0+1 \\ \end{Bmatrix} \ , \end{aligned}$$

where *q*(*x*) is a third-order polynomial fixed uniquely by the requirement that *f*(*x*) and its first derivative be continuous functions. A typical form of the function defined by Eq. (9) is shown in Fig. 3. Our choice of *f*(*x*) is suggested by Eq. (8). As one can see from this expression, if for large *n*, the $Q_n$’s form a geometric sequence such that $Q_{n+1}/Q_n=b<1$, then the choice $\beta = b-1$ in Eq. (9) will reproduce such behavior that, (provided $|\beta +1|<1$) guarantees the existence of a steady state solution of the Kolmogorov equation. Similarly, if for small *n*, the $Q_n$’s behave so that $Q_{n+1}/Q_n=a$, we might use $\alpha = a-1$ in Eq. (9). Finally, the choice of the parameter $x_0$ fixes the position of the maximum of the distribution. We have thus three parameters, $\alpha$, $\beta$, and $x_0$, in Eq. (9) which we will consider as fitting parameters for the trial form of *f*(*k*). Results of the three-parameter fits, based on Eq. (9), are shown in Fig. 1.

## Discussion

Our results, presented in Fig. 1, show that the SFA results obtained using the procedure described in the section “Methods” below, and the *ab initio* results obtained by solving the TDSE (3) with the QED Hamiltonian, agree reasonably well. The three-parameter fitting procedure, based on the ’birth-death’ model we described above, gives a considerably better agreement with the calculated distributions $Q_n$. The relatively small deviation between the TDSE and the SFA results can be due to the fact that SFA neglects effect of the atomic potential on the ionized electron. For systems with Coulomb potential, for instance, SFA can give predictions for ionization probabilities which are a few orders of magnitude smaller than the results of TDSE calculations, unless special care is taken to take into account Coulomb interaction^8^. For systems with short range interactions TDSE and SFA usually agree better, but we can still expect some differences for a potential of a small but finite radius of the order of one atomic unit, like the potential *V*(*r*) we use. This can be seen if we note that the physical results do not depend on the gauge used for the description of the electromagnetic field. SFA results obtained using the commonly used length and velocity gauges, on the other hand, may differ^8^, thereby implying presence of an intrinsic, albeit possibly relatively small, error. SFA becomes a gauge-independent theory only for the zero-range potential^8^.

For the reader’s convenience we summarize briefly the basic premises on which this model was based. We first demonstrated that the non-diagonal elements of the reduced density matrix describing the field vanish with time. We believe this phenomenon presents an interesting example of the decoherence process which may occur when we follow the evolution of a subsystem interacting with its environment. The subsystem in question and the environment are, in our case, the field and the atom, respectively. Looking at the atom as the environment is certainly not a conventional way to look at the ionization process. We saw, however, that the mechanism leading to the decay of the non-diagonal matrix elements of the photon density matrix is the same as in the more conventional examples of the decoherence phenomenon, e.g., a subsystem interacting with a collection of harmonic oscillators^41,49,50^. Different environment states in the entangled subsystem+environment state become approximately orthogonal with time. This leads to the suppression of the non-diagonal elements of the reduced density matrix describing the subsystem^41,42^. The source of this approximate orthogonality in the present case is the fact that, for large enough times, different atomic vectors in the expansion Eq. (4) become well separated in energy.

As for the diagonal matrix elements of the reduced photon density matrix (5), we proposed a simple model which pictures absorption and emission of photons in the process of strong-field ionization as a ’birth-death’ process. An assumption we made was the Markovian character of the process. For the related problem of the one-mode electromagnetic field in a cavity, the master equation, governing evolution of the field, can be cast in a form^39^that is reminiscent of the Kolmogorov equation describing the birth-death stochastic models. We adopted this equation as the equation governing the evolution of the reduced photon density matrix for the strong field ionization process. We were interested in the steady state solution of the Kolmogorov equation, to which $Q_n(t)$ tends in the limit $t\rightarrow \infty$. As we noted, the steady state solution does not always exist. For instance, we would not have such a solution for a pure birth process (all $\mu _k=0$ in Eq. (7)). If, for example, we set all coefficients $\mu _k$ to zero in Eq. (7) and assume that all coefficients $\lambda _k$ have equal values, $\lambda _k =\lambda$, then the solution of Eq. (7) would be the Poisson distribution^48^ $Q_n(t)= e^{-\lambda t}(\lambda t)^n/n!$ which does not have a non-trivial steady state limit for $t\rightarrow \infty$. That photon absorption distributions are distinctly non-Poissonic has been noticed in the literature^7,38,51^. In framework of our model, based on the Kolmogorov equation, the non-Poissonic character of the distribution of absorbed photons is just a consequence of the fact that the Poisson distribution is not a steady state distribution; $Q_n$’s in this case explicitly depend on time.

## Conclusions

We studied evolution of the quantum system consisting of atom interacting with quantized electromagnetic field. The study was based on the numerical solution of the time-dependent Schroödinger equation driven by the QED Hamiltonian. Use of the QED picture allowed us to study probability distribution $Q_n$ of photons absorbed in the tunneling regime of strong field ionization.

We proposed a statistical model based on the view of ionization as a stochastic birth-death process. In framework of this model the distribution $Q_n$ can be interpreted as a steady state solution of the Kolmogorov equation to which absorbed photons distribution tends in the limit of large times. Making an assumption about the behavior of the ratio of the birth and death rates $\lambda _{k-1}/\mu _k$ of the stochastic process encapsulated by Eq. (9), with three parameters considered as fitting parameters, we obtained results presented in Fig. 1. The Figure shows reasonable agreement of the model distribution given by the steady state solution of Eq. (7) and the results of the *ab initio* numerical calculation of the distribution of absorbed photons.

## Methods

### Solution of the TDSE

We will use a co-ordinate representation for the vectors $|f_N(t)\rangle$ in the expansion Eq. (4). We will omit, therefore, the Dirac notation for $|f_N(t)\rangle$ and will simply write $f_N({{\varvec{r}}},t)$, understanding them as functions of the spatial coordinates and time. To solve the TDSE (3) we represent $f_N({{\varvec{r}}},t)$ as (we employ the geometry imposed by the field geometry with the polarization vector along the *z*-direction):

10 $$\begin{aligned} f_N({{\varvec{r}}},t) = \sum \limits _{l=0}^{l_\text{max}} f_{Nl}(r,t) Y_{l0}({{\varvec{n}}}) \ . \end{aligned}$$

The radial variable *r* is treated by discretizing the TDSE on a grid with a step-size $\delta r=0.1$ a.u. in a box of size $R_\text{max}$. Upon substituting expansions (4) and (10) into Eq. (3), projecting the result on vectors $Y_{l_i0}({{\varvec{n}}}) \otimes |N_i\rangle$ with different $l_i$, $N_i$, and computing the arising matrix elements, we obtain a system of coupled evolution equations for the radial functions $f_{Nl}(r,t)$. This system of coupled equations is solved using a matrix iteration method^52^. This procedure, in fact, is quite similar to the procedure employed for the solution of the ordinary atomic TDSE reported previously^53–55^.

**Figure 4:**
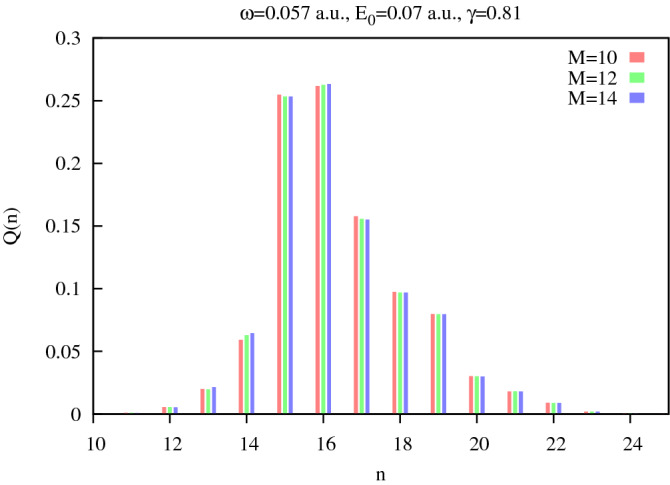
Absorbed photons probability distributions $Q_n$, obtained for different propagation intervals in Eq. (11) for an effective field strength of 0.07 a.u.

The computational cost of realization of the strategy based on the expansions (4), (10) depends on the values of the parameters $n_1$, $n_2$ in Eq. (4), parameter $l_\text{max}$ in Eq. (10), and parameter $R_\text{max}$ defining the size of the box. The parameters $n_1$ and $n_2$ should be roughly of the order of the maximum number of photons which can be absorbed (parameter $n_1$) or emitted (parameter $n_2$) during the evolution. To choose parameters $l_\text{max}$ and $R_\text{max}$ properly, we relied on the experience gained in solving ordinary atomic TDSE for the classical field with field intensity related to the photon number $N_0$ according to the relation we gave above. For instance, for the solution of Eq. (3) for the equivalent field strength of $E_0=0.1$ a.u. (corresponding intensity of $3.51\times 10^{14}$ W/cm$^2$), we used $R_\text{max}=1500$ a.u., $l_\text{max}=50$, $n_1=n_2=50$. The choice of $R_\text{max}$ also depends, of course, on the duration of the time interval over which we solve the TDSE (3). As mentioned above, for the majority of the calculations we report, this interval was (0, 12*T*), where $T=2\pi /\omega$, with an optical cycle corresponding to the driving frequency $\omega =0.057$ a.u. Solving the TDSE, we introduce a cutoff envelope function *g*(*t*), so that the expression for the vector potential used for the solution of the TDSE (3) is $g(t)\hat{{{\varvec{A}}}}({{\varvec{r}}},t)$ where $\hat{{{\varvec{A}}}}({{\varvec{r}}},t)$ is the operator given by Eq. (1), and the envelope function was chosen as:

11 $$\begin{aligned} g(t)= \sin ^2{\left( {\pi t\over MT}\right) } \qquad t\in (0,MT) \ , \end{aligned}$$

where (0, *MT*) is the time interval on which the TDSE has been solved. Introduction of the adiabatic turning on and off of the interaction is a commonly-employed procedure in quantum field theory, used e.g., for the construction of S-matrix^56^. In our case, the introduction of the envelope function is also necessary for computational reasons. We cannot propagate the TDSE numerically on very long time intervals, and we wish to avoid the effects of a sudden turning on and off of the interaction. We must ensure, of course, that the envelope function we use is indeed adiabatic, i.e., the results we obtain are not affected by the choice of the duration of time interval (0, *MT*) on which we propagate the TDSE. We presented an extensive study of this question in the work^38^, where we solved the TDSE for the system atom+photon field using a semi-classical description of the quantized electromagnetic field, based on an approach proposed in^37^. We showed in that work that the results for the photon number distribution are not affected by the particular choice of envelope function, as long as the interval on which the TDSE is propagated is sufficiently long. To show that this conclusion remains valid in the present case, when we solve the fully quantum TDSE using expansion (4), we show, in Fig. 4, the absorbed photons probability distributions $Q_n$, obtained for different pulse durations for an effective field strength of 0.07 a.u.

### Estimate of the photon density matrix based on the SFA

To estimate the elements of the reduced density matrix (5) describing the photon field, we can use a semi-classical method for the description of the quantized electromagnetic field proposed in^37^. In the framework of this procedure, the atomic Hilbert space vector $|f_N(t)\rangle$ in the expansion (4) can be found as a Fourier transform:

12 $$\begin{aligned} f_N({{\varvec{r}}},t) = {1\over {2\pi }}\int \limits _0^{2\pi } \Psi ({{\varvec{r}}},t,\theta ) e^{-im\theta }\ d\theta \ , \end{aligned}$$

where $m=N-N_0$, and the vector $|\Psi (t,\theta )\rangle$ belonging to the atomic Hilbert space is a solution of the time-dependent Schrödinger equation:

13 $$\begin{aligned} i{\partial \Psi ({{\varvec{r}}},t,\theta )\over \partial t}= \left( \hat{H}_\text{atom} + {1\over c}\hat{{{\varvec{p}}}}{{{\varvec{A}}}(t,\theta )} + {1\over 2c^2}{{{\varvec{A}}}(t,\theta )}^2 \right) \Psi ({{\varvec{r}}},t,\theta ) \ , \end{aligned}$$

with an initial condition such that $\Psi ({{\varvec{r}}},t,\theta )$ is the ground atomic state at $t=0$, and ${{{\varvec{A}}}(t,\theta )}$ is a classical field:

14 $$\begin{aligned} {{{\varvec{A}}}}(t,\theta )= \sqrt{2N\pi c^2\over \omega V} g(t) {{\varvec{e}}}_z \left( e^{-i\omega t-i\theta } + c.c. \right) = g(t){{\varvec{e}}}_z A_0\cos {(\omega t + \theta )} \ , \end{aligned}$$

with the same effective amplitude $\displaystyle A_0= \sqrt{8N_0\pi c^2\over \omega V}$ as the quantum electromagnetic field (1), and the same envelope function *g*(*t*) we used above in the fully quantum calculation. The effect of the quantum nature of the field in the semi-classical approach^37^ reveals itself through the presence of the classical phase $\theta$, uniformly distributed in the interval $(0,2\pi )$. The appearance of the uniformly-distributed classical phase can be traced back^37^ to the fact that the phase of the field is completely undetermined in the Fock state of the field^36^. By computing the Fourier transform of the solution of the classical TDSE (13), as prescribed by Eq. (12), we can find the components $|f_N(t)\rangle$ in the Eq. (4).

To use this recipe, we need an analytic estimate for the solution of the classical TDSE (13). This estimate can be obtained using the SFA. In framework of this approximation, the solution to Eq. (13), satisfying the condition that the atom is in the ground atomic state $\phi _0({{\varvec{r}}})$ at $t=0$ can be written as^7,8^:

15 $$\begin{aligned} \Psi ({{\varvec{r}}},t,\theta )= \phi _0({{\varvec{r}}})e^{-i\varepsilon _0 t} + \int a({{\varvec{k}}},t,\theta ) { e^{i{{\varvec{k}}}\cdot {{\varvec{r}}}}\over (2\pi )^{3/2}} \ , \end{aligned}$$

where $\varepsilon _0$ is the ground state energy, and $a({{\varvec{k}}},t,\theta )$ are the SFA ionization amplitudes given in the velocity gauge which we use by the expression^7,8^:

16 $$\begin{aligned} a({{\varvec{k}}},t,\theta )= -i\int \limits _0^t \tilde{\phi }_0({{\varvec{k}}}) \left( {k^2\over 2}+ {{\varvec{k}}}\cdot {{\varvec{A}}}(\tau ,\theta )\right) \exp {\left\{ -i\int \limits _{\tau }^t {\left( {{\varvec{k}}}+{{{\varvec{A}}}}(x,\theta )\right) ^2\over 2}\ dx -i \varepsilon _0\tau \right\} } \ d\tau \ , \end{aligned}$$

where the classical vector potential ${{\varvec{A}}}(\tau ,\theta )$ is given by Eq. (14), and $\tilde{\phi }_0({{\varvec{k}}})$ is the Fourier transform of the initial state wave-function. Using Eq. (16) we can compute the quantity $\displaystyle \tilde{a}({{\varvec{k}}},t,m)=\int \limits _0^{2\pi } a({{\varvec{k}}},t,\theta ) e^{-im\theta } \ {d\theta \over 2\pi }$. We will need only the elements of the photon density matrix with $N_1< N_0$, $N_2<N_0$ (corresponding to the states of the field with at least one photon absorbed by the atom), where the term $\phi ({{\varvec{r}}})e^{-i\varepsilon _0 t}$ in Eq. (15) does not contribute. Using Eq. (12) and Eq. (15), we obtain, for these elements of the photon density matrix in Eq. (5):

17 $$\begin{aligned} \rho _F^{N_1N_2}(t)= \langle f_{N2}(t)|f_{N1}(t)\rangle =\int \tilde{a}({{\varvec{k}}},t,m_2)^*a({{\varvec{k}}},t,m_1) \ d{{\varvec{k}}}\ , \end{aligned}$$

where $m_1=N_1-N_0$, $m_2=N_2-N_0$. All the integrals in Eq. (17), Eq. (12), Eq. (15) were computed numerically. The calculation is quite straightforward and we will not dwell upon its details.

### Justification of the use of Kolmogorov equation (7) for the diagonal elements of the reduced photon density matrix

In the present section, we describe in more detail the reasoning which led us to the assumption that the evolution of the diagonal elements of the reduced photon density matrix can indeed be described by the Kolmogorov equation (7). We cannot provide a mathematically rigorous proof of this statement. Indeed, the task we have at hand is the description of the irreversible behavior of a system interacting with a reservoir, which is a notoriously difficult problem. It can be solved in some instances when simplifying assumptions about the reservoir can be made, e.g., the assumption that the reservoir is affected very little by the system and that it remains in a state of thermal equilibrium during the process. Such an assumption was made in^39^; it allows one to obtain a Kolmogorov-type equation describing the evolution of the reduced photon density matrix for the field interacting with the cavity. This assumption amounts to postulating that the reservoir is sufficiently large (i.e. contains many degrees of freedom) and that typical relaxation times of the reservoir are much faster than the typical time interval over which the photon density matrix changes appreciably. We can hardly use these assumptions in the present case, where the role of the reservoir is played by the atomic system. We cannot, therefore, use this line of argument.

We will, instead, present some arguments of heuristic character, based primarily on numerical evidence. First, we note that the three-term structure of Eq. (7) appears quite naturally in the quantum mechanical equation for the evolution of the diagonal elements of the photon density matrix. The latter can be obtained from the expression (5) for the density matrix elements. We will employ, as above, the notation $P_n(t)= \hat{\rho }_F^{{N_0-n},{N_0-n}}$ for the diagonal elements of the reduced photon density matrix. We can use the above-mentioned fact that, in the semi-classical method proposed in^37^ (which is an excellent approximation for the field parameters we consider), the atomic Hilbert space vector $|f_N(t)\rangle$ in the expansion (4) is a Fourier transform (12) of the solution $\Psi ({{\varvec{r}}},t,\theta )$ of the time-dependent Schrödinger equation (13) with the classical vector potential ${{\varvec{A}}}(t,\theta )$ given by the Eq. (14). Using Eq. (12), Eq. (13), and expressing $\Psi ({{\varvec{r}}},t,\theta )$ in terms of $|f_N(t)\rangle$ by using Fourier transform inverse to Eq. (12), we obtain:

18 $$\begin{aligned} {\partial f_N({{\varvec{r}}},t)\over \partial t}=-{i\over {2\pi }}\int \limits _0^{2\pi }\sum \limits _{m_1} \hat{H}(t,\theta )f_{N_1}({{\varvec{r}}},t) e^{i(m_1-m)\theta } \ , \end{aligned}$$

where $m_1=N_1-N_0$, and $\hat{H}(t,\theta )$ is the Hamiltonian operator on the right hand side of the Eq. (13). Projecting Eq. (18) on $f_{N}({{\varvec{r}}},t)$ and using the explicit expression for the Hamiltonian (13) and vector potential (14), we obtain for the time derivative of $P_n(t)=\langle f_{N_0-n}(t)|f_{N_0-n}(t)\rangle$:

19 $$\begin{aligned} {dP_n(t)\over dt}\approx A_0 g(t) \mathfrak {I}\left( \langle f_N(t)|\hat{p}_z|f_{N-1}(t)\rangle e^{i\omega t}+ \langle f_N(t)|\hat{p}_z|f_{N+1}(t)\rangle e^{-i\omega t} \right) \ , \end{aligned}$$

where $A_0$ and *g*(*t*) are the amplitude and the envelope function in Eq. (14), $\mathfrak {I}(z)$ stands for the imaginary part of a complex *z*. Deriving Eq. (19), we neglected terms proportional to the overlaps $\langle f_N(t)|f_{N_1}(t)\rangle$ with $N\ne N_1$ which give us non-diagonal elements of the photon density matrix. As we have seen, these vanish with time due to the decoherence process. Eq. (19) is not yet of the form of the Kolmogorov equation Eq. (7) since it involves matrix elements of the momentum operator calculated with amplitudes $f_N(t)$, and not the overlaps of the $f_N(t)$ themselves, which we need in order to express the right-hand-side of Eq. (19) in terms of the diagonal density matrix elements $P_n(t)$. Formally, we can write Eq. (19) in the form of Eq. (7) by rewriting the matrix elements in Eq. (19) as:

20 $$\begin{aligned} \langle f_N(t)|\hat{p}_z|f_{N-1}(t)\rangle = a(t) P_n(t) {\langle f_N(t)|\hat{p}_z|f_{N-1}(t)\rangle \over \langle f_N(t)|f_{N}(t)\rangle } + b(t) P_{n+1}(t) {\langle f_N(t)|\hat{p}_z|f_{N-1}(t)\rangle \over \langle f_{N-1}(t)|f_{N-1}(t)\rangle } \ , \end{aligned}$$

and similarly for the second matrix element in Eq. (19). In Eq. (20) *a*(*t*) and *b*(*t*) are functions of time satisfying $a(t)+b(t)=1$, but are otherwise arbitrary. In order for Eq. (20) to be of the Kolmogorov form (7), these functions must be chosen so that the coefficients with $P_n(t)$, $P_{n-1}(t)$ and $P_{n+1}(t)$ resulting upon substituting Eq. (20) (and an analogous expression for the matrix element $\langle f_N(t)|\hat{p}_z|f_{N+1}(t)$) in Eq. (19)) be approximately time-independent. We can provide only numerical evidence indicating that such a choice is indeed possible, and that the diagonal photon density matrix elements $P_n(t)$ calculated in the present work do approximately satisfy a Kolmogorov-type equation. To show this, let us introduce the functions $\tilde{P}_n(t)=P_n(t)/P_n$ where $P_n$ are the constant values which $P_n(t)$ assume at the end of the pulse. If we assume that $P_n$ are indeed stationary limiting values of the random birth-death process described by Eq. (7), then, from Eq. (8), we must have: $P_{n+1}/P_{n}=\lambda _{n}/\mu _{n+1}$. Using this relation and the Kolmogorov equation (7), one obtains the following equation which $\tilde{P}_n(t)$ must satisfy if our basic assumptions about the statistical character of the process are correct:

21 $$\begin{aligned} {d\tilde{P}_n(t)\over dt}= \mu _{n}\tilde{P}_{n-1}(t)+ \lambda _{n}\tilde{P}_{n+1}(t)- (\lambda _{n}+ \mu _{n})\tilde{P}_n(t) \ . \end{aligned}$$

**Figure 5:**
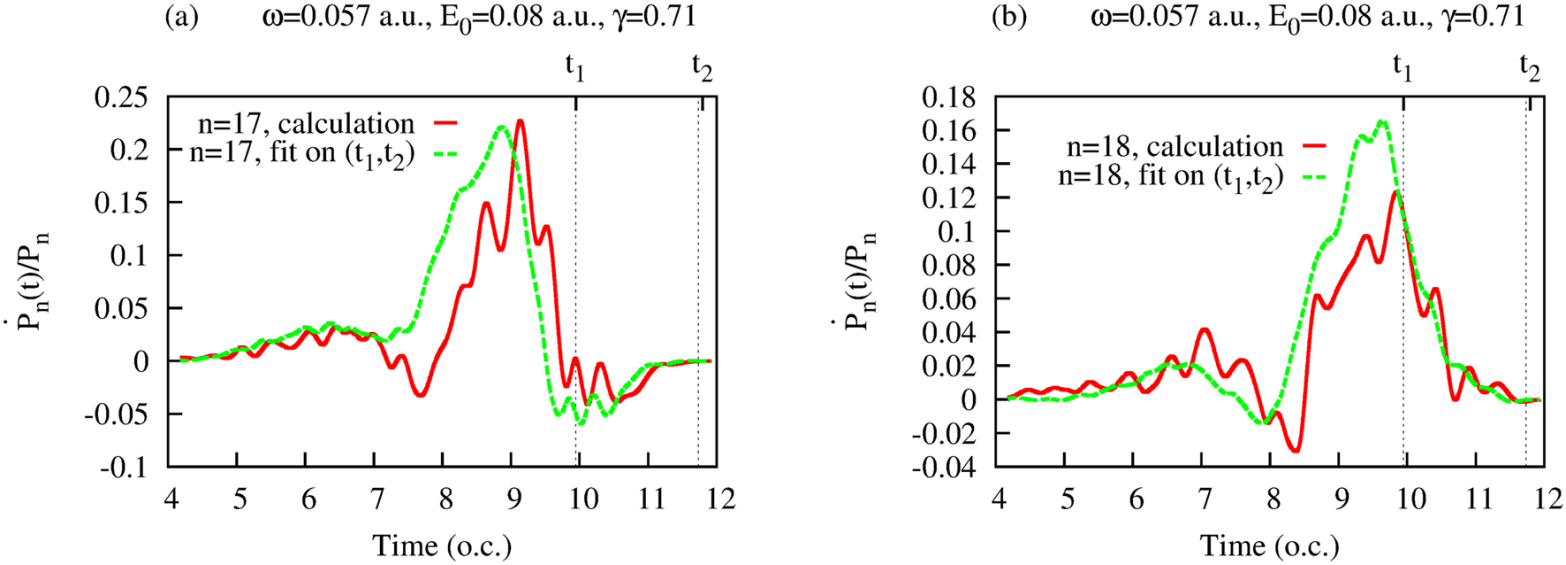
(Color online) Results of the fit based on the Eq. (22) for $\dot{P}_n(t)/P_n$ for $n=17$ and $n=18$. Dashed vertical lines indicate the boundaries of the interval $(t_1,t_2)$ on which the fitting procedure was applied.

**Figure 6:**
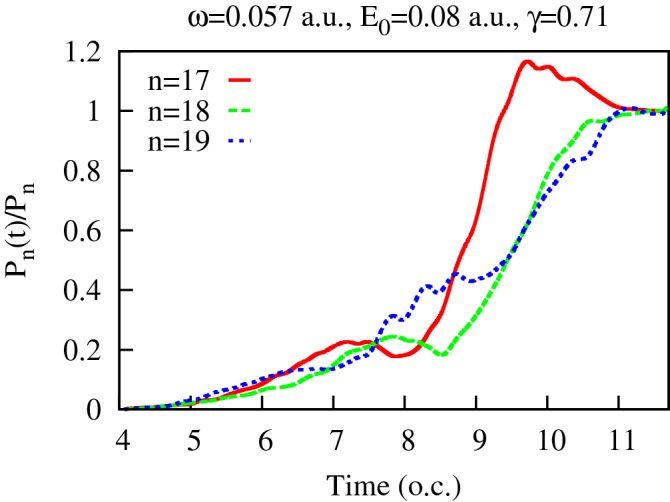
(Color online) Functions $P_n(t)/P_n$ for $n=17$, 18 and 19.

This equation is somewhat easier to handle numerically than Eq. (7) since we have only $\mu _n$ and $\lambda _n$ with the same *n* on the right-hand-side. We can check now, if the $\tilde{P}_n(t)$ we obtain from our numerical calculations indeed satisfy equation Eq. (21) with some coefficients $\mu$ and $\lambda$. A straightforward way to proceed is to use a least-squares fit, taking as an input the computed values of $\tilde{P}_n(t)$ and their derivatives, and using Eq. (21) as the fitting expression. More specifically, we form the functional:

22 $$\begin{aligned} S(\mu _n,\lambda _n)= \sum \limits _{t_i\in (t_1,t_2)} \left( {d\tilde{P}_n(t_i)\over dt}- \mu _{n}\tilde{P}_{n-1}(t_i)- \lambda _{n}\tilde{P}_{n+1}(t_i)+ (\lambda _{n}+ \mu _{n})\tilde{P}_n(t_i) \right) ^2 \ , \end{aligned}$$

where the values of $P_n$ and its derivative are computed on the interval $(t_1,t_2)$, and we seek the minimum of the functional (22) with respect to variations of the parameters $\mu _n$,$\lambda _n$. The results of this procedure for a particular field strength of 0.08 a.u. and particular values of $t_1$, $t_2$ defining the interval on which the fitting procedure is applied, are shown in Fig. 5 for $n=17$ and $n=18$ (which are the values of *n* for which the distribution of the absorbed photons have a maximum for this intensity, as Fig. 1 shows). One can see that the fit based on Eq. (22) reproduces the correct behavior of the derivative ${d\tilde{P}_n(t)\over dt}$ fairly well. That this fact is not entirely trivial can be seen from Fig. 6, where we show the functions $\tilde{P}_{n}(t)$ appearing on the right-hand-side of Eq. (21) for $n=18$. If the functions $\tilde{P}_{n}(t)$ were simple (e.g., monotonic) functions of time, the success of the fitting procedure (22) could be regarded as a mere coincidence. This is not the fact, however. As Fig. 6 shows, the functions $\tilde{P}_{n}(t)$ are rather complicated functions of time. Even more important, perhaps, is the fact that, to obtain the results shown in Fig. 5, we used the interval $(t_1,t_2)$ at the end of the pulse. As one can see form the Figure, the fitting expression (22) with the coefficients $\mu _n$,$\lambda _n$ obtained for this interval proves relatively accurate even for *t*-values lying well outside the interval $(t_1,t_2)$. This, in our opinion, shows that the fitting formula (22) indeed captures essential features of the behavior of the diagonal matrix elements of the reduced photon density matrix.

We can also see that Eq. (21) approximately describes ionization dynamics by converting the difference equation into a differential one. Let us consider the function $\tilde{P}(n,t)$, which for integer *n* coincides with $\tilde{P}_{n}(t)$. Then, from Eq. (21), one can obtain a partial differential equation that $\tilde{P}_{n}(t)$ should approximately satisfy:

23 $$\begin{aligned} {\partial \tilde{P}(n,t)\over \partial t} \approx {\partial \tilde{P}(n,t)\over \partial n} (\lambda _n-\mu _n) \ . \end{aligned}$$

**Figure 7:**
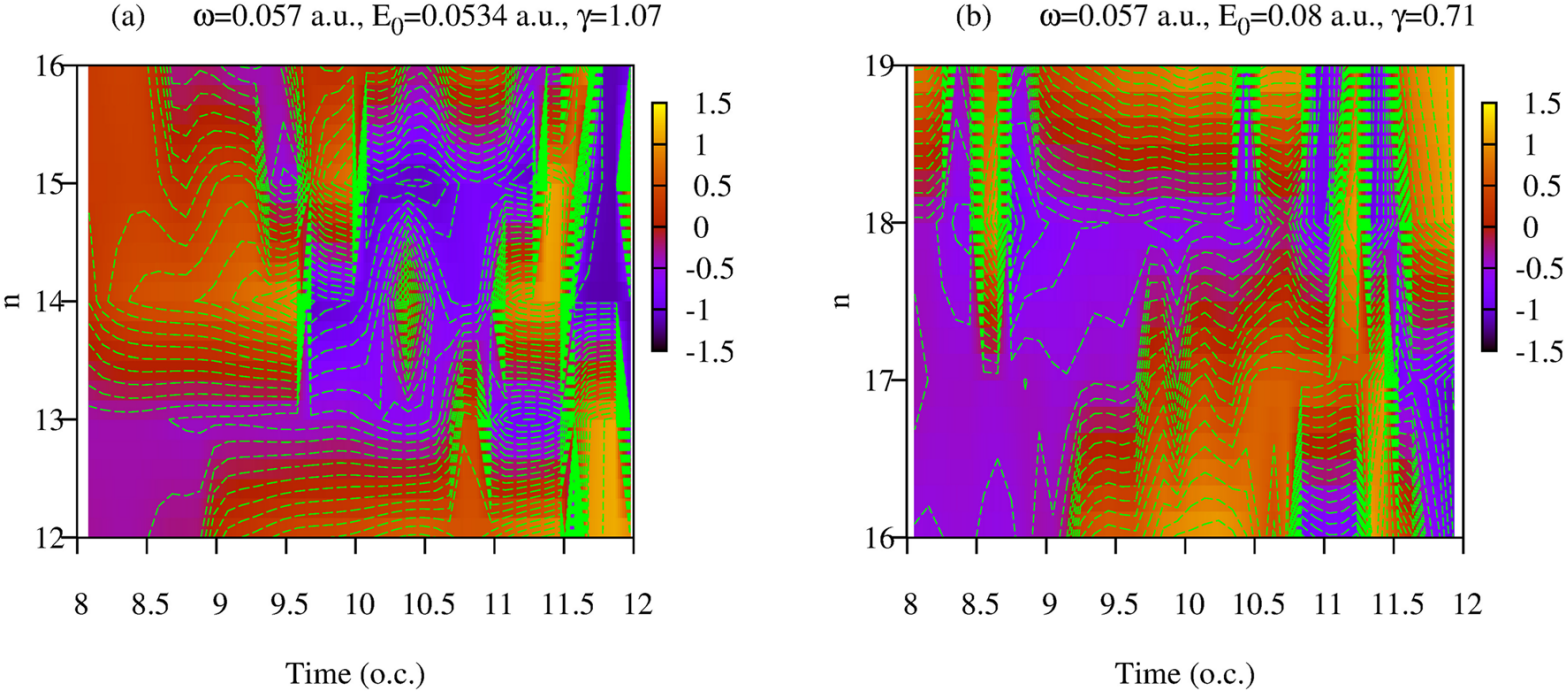
(Color online) Lines of constant elevation for the function $h(t,n)=\arctan \left\{ {\partial \tilde{P}(n,t)\over \partial t}/{\partial \tilde{P}(n,t)\over \partial n}\right\}$.

A consequence of this equation is that the ratio ${\partial \tilde{P}(n,t)\over \partial t}/{\partial \tilde{P}(n,t)\over \partial n}$ is a function of *n* only. Fig. 7 shows lines of constant elevation of the function $h(t,n)=\arctan \left\{ {\partial \tilde{P}(n,t)\over \partial t}/{\partial \tilde{P}(n,t)\over \partial n}\right\}$ in the $(t,n)-$ plane (we use arctangent of the ratio to make *h*(*t*, *n*) vary within finite limits). If Eq. (23) is approximately valid, the lines of constant elevation of *h*(*t*, *n*) should be the lines of constant *n*. This is indeed approximately the case, as can be seen from Fig. 7. It shows that lines of constant elevation of *h*(*t*, *n*) are indeed lines $n\approx \mathrm{const}$ everywhere in the (*n*, *t*)-plane apart from some regions, which are in fact the neighborhoods of points where ${\partial \tilde{P}(n,t)\over \partial n}$ has zeros. When deriving Eq. (23), we approximated finite differences with first-order partial derivatives. This approximation fails in the vicinity of zeros of ${\partial \tilde{P}(n,t)\over \partial n}$. Therefore, the deviation of the lines of the constant elevation from $n\approx \mathrm{const}$ in the vicinity of zeros of ${\partial \tilde{P}(n,t)\over \partial n}$ is to be expected. We believe, therefore, that Fig. 7 provides good evidence in favor of the approximate validity of Eq. (23). Assuming that this equation is valid, we can retrace the steps we used to derive it and obtain the Kolmogorov equation Eq. (7) for the diagonal elements of the reduced photon density matrix.
